# Blood transcriptome analysis in a buck-ewe hybrid and its parents

**DOI:** 10.1038/s41598-019-53901-z

**Published:** 2019-11-25

**Authors:** Clemens Falker-Gieske, Christoph Knorr, Jens Tetens

**Affiliations:** 1 Department of Animal Sciences, Georg-August-University, Göttingen, Germany; 2 Center for Integrated Breeding Research, Georg-August-University, Göttingen, Germany

**Keywords:** Genetic hybridization, Gene expression, Gene ontology, Animal breeding, Functional clustering

## Abstract

Examples of living sheep-goat hybrids are rare, mainly due to incorrect chromosome pairing, which is thought to be the main cause for species incompatibility. This case represents the first report of a buck-ewe hybrid and the first mammalian hybrid to be analyzed with next generation sequencing. The buck-ewe hybrid had an intermediate karyotype to the parental species, with 57 chromosomes. Analysis of the blood transcriptomes of the hybrid and both parents revealed that gene expression levels differed between the hybrid and its parents. This could be explained in part by age-dependent differences in gene expression. Contribution to the geep transcriptome was larger from the paternal, compared to the maternal, genome. Furthermore, imprinting patterns deviated considerably from what is known from other mammals. Potentially deleterious variants appeared to be compensated for by monoallelic expression of transcripts. Hence, the data imply that the buck-ewe hybrid compensated for the phylogenetic distance between the parental species by several mechanisms: adjustment of gene expression levels, adaptation to imprinting incompatibilities, and selective monoallelic expression of advantageous transcripts. This study offers a unique opportunity to gain insights into the transcriptome biology and regulation of a hybrid mammal.

## Introduction

A number of case studies of the living hybrid offspring between sheep (*Ovis aries*, *O. aries*) and goats (*Carpra hircus*, *C. hircus*) have been described^1–5^. However, there has only been one reported case of a buck-ewe hybrid (geep)^6^ and all other cases have involved the mating of goats with rams. A restricted species incompatibility due to reproductive isolation is most likely the reason for the rareness of buck-ewe hybrids. Despite the genetic similarity of the parental species, the hybrid organism has to compensate for genomic diversities^7^. Pauciullo *et al*. showed that the geep had an intermediate karyotype with 57 chromosomes, whereas the ewe had 54 and the buck had 60 chromosomes^6^. Since parthenogenic sheep embryos exhibit growth retardation and early embryonic death, genomic imprinting is considered to be essential for ruminant development^8^ and is most likely aberrant in the geep compared to the parental species^9^. Genes such as *IGF2* and *PEG1/MEST* are expressed from the paternal genome in mice, humans, and in sheep^8,10,11^, whereas maternally imprinted genes in sheep include *H19*, *IGF2R*, *GRB10* and *p57*^*KIP*^ ^12^. A hybrid ruminant individual presents the opportunity to gain further insights into distinct maternal and paternal contributions to the offspring’s transcriptome and genetic imprinting, since clear distinctions between the paternal and maternal gene sequences exist. In the follow up study to Pauciullo *et al*. (2016)^6^ presented here, we analyzed the blood transcriptomes of the geep and its parents, which to our knowledge is the first mammalian hybrid to be studied with next generation sequencing. RNA from whole blood of all three animals was sequenced on an Ion Torrent platform. Four widely used alignment methods were compared to map sequencing reads to the latest sheep and goat reference genome assemblies. We analyzed the blood transcriptomes of the geep and its parents comparatively and found that the geep had considerably less transcripts in common with its parents among the most highly expressed genes. The number of common genes between the parents was higher, which is explainable by age-dependent gene expression. Furthermore, the transcriptome overlap was larger between the geep and the goat than the geep and the sheep. Genes that were commonly expressed between the geep and goat are enriched in enzyme activity and defense mechanisms, whereas commonly expressed geep and sheep genes play a role in nucleic acid and ion metabolism. Additionally, we performed variant calling to make use of the full sequencing depth. Variants that were found to be expressed alternatively monoallelic in the founders were retained for further analyses. This enabled us to the draw the conclusions that goat contribution to the geep transcriptome was higher and that the geep compensates for probable deleterious variant effects with biallelic expression when monoallelic expression was to be expected. In conclusion, this study presents the first comprehensive analysis of the complete transcriptome of a higher hybrid mammal and serves as a basis for a deeper understanding of evolutionary mechanisms that involve hybridization.

## Results

### Alignment to reference genomes

**Figure 1:**
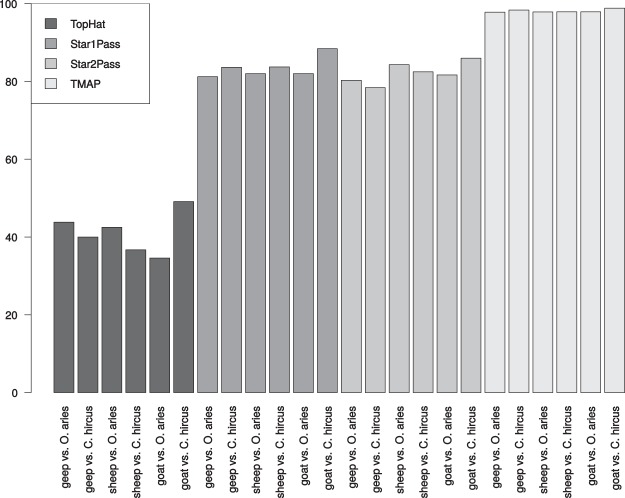
Comparison of short sequencing read mapping software packages. Mapping efficiencies of TopHat, Star1pass, Star2pass, and TMAP of geep, sheep and goat RNA sequencing reads against the *O. aries* and *C. hircus* reference genomes were visualized. Mapping efficiencies varied from 34.6% to 98.3% depending on the software packages.

**Figure 2:**
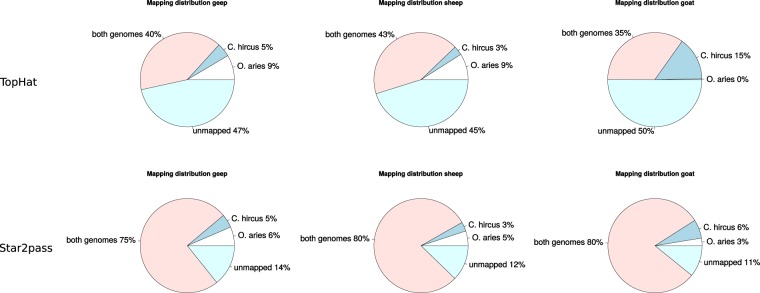
Species discrimination of the two most promising mapping algorithms, TopHat and Star2pass. The distribution of reads mapped against each reference genome (*O. aries* and *C. hircus*) by TopHat and Star2pass was evaluated. Mapping efficiencies of Star2pass were higher in comparison to TopHat, whereas TopHat performed better with respect to species discrimination. Since Star2pass provided the best trade-off between mapping efficiencies and species discrimination it was used for all further analyses.

To elucidate, which mapping software is best suited for a hybrid mapping approach, we tested the mapping efficiencies of four different software packages (Fig. 1). TopHat performed best in respect to species discrimination (Fig. 2) whilst mapping efficiencies were low. Star2pass had acceptable mapping efficiencies with an acceptable capability to discriminate between species. TMAP, which is optimized for the mapping of Ion Torrent reads, exhibited mapping efficacies close to 100% without any species discrimination. Geep sequencing reads that uniquely map to either reference genome were identified as described in material and methods. Of 90,701,679 geep sequencing reads 14,011,831 (15.4%) reads could be uniquely assigned to the *O. aries* reference assembly and 15,371,814 (16.9%) reads to the *C. hircus* reference assembly using Star2pass mapped reads as input data. 14% of the reads did not map to either genome and the remaining reads had to be discarded because the alignment scores were identical. Due to the low mapping efficiencies the TopHat alignments were not further processed.

### Differential expression and transcriptome comparison

**Figure 3:**
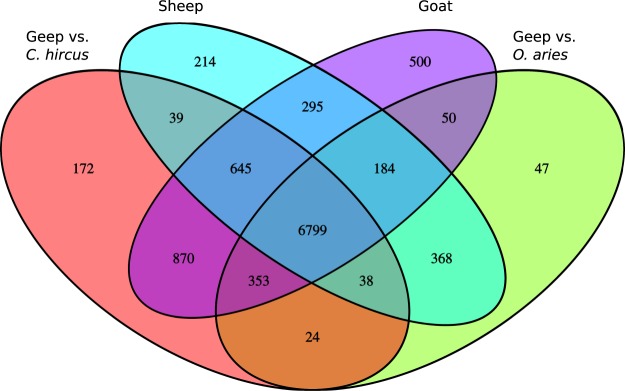
Venn diagram visualizing overlapping transcripts between geep, sheep and goat transcritpomes with a FPKM value >1. The numbers in the fields describe the number of transcripts that the four analyzed groups of expressed genes have in common at a given intersection.

Transcripts with a Fragments Per Kilobase Million (FPKM) value >1 were retained and the number of transcripts discovered for each dataset is summarized in Supplementary Table S1. The complete Cufflinks output is summarized in Supplementary Table S2. For all downstream analyses, genes of uncertain function were removed since they provide no useful information for species comparison. The 10 genes with the highest expression levels from each dataset are shown in Table 1. The parents show a common expression of 80 genes among the 100 highest expressed genes, whereas 61 genes are commonly expressed in all three animals (more detailed summary in Supplementary Table S3). Common genes between animals among the top 100 expressed genes are listed in Supplementary Table S4. By assigning each read from the geep RNAseq dataset uniquely to either the *O. aries* or the *C. hircus* reference genome, we were able to perform a transcriptome comparison (Fig. 3). Comparatively few (n = 24) geep transcripts were assigned to both genomes, which serves as an internal control for the functionality of the pipeline for the generation of unique sequencing reads. 870 geep transcripts stem from the buck and 368 from the ewe. The results for a functional annotation clustering of the two groups are shown in Supplementary Table S5. To compile a list of genes that are expressed in an age-dependent manner, genes uniquely expressed by the geep or the founder animals were cross referenced with genes that were found to be age-dependently expressed in human blood^13^. The results are summarized in Supplementary Table S6. Over represented pathways are shown in Table 2. Genes uniquely expressed by the geep, or those that overlapped with one parent only, are summarized in Supplementary Table S7. By cross-referencing genes from the geep transcriptome that mapped to one reference genome only (genes only annotated in one species were excluded) using the geneimprint database (http://www.geneimprint.com, accessed May 2019), we found 14 matches (Table 3). Of these, 9 genes do not match the parental origin found in the database. A pathway analysis with PANTHER revealed that the number of genes involved in the gonadotropin-releasing hormone receptor pathway (P06664) is elevated in the group of genes that the geep and the sheep express (geep-sheep intersection 16 out of 870 genes, geep-goat intersection 3 out of 368 genes). Furthermore, genes associated with inflammation mediated by chemokine and cytokine signaling pathway (P00031) were elevated in the group of genes that only the geep and the goat express (geep-sheep intersection 7 out of 870 genes, geep-goat intersection 6 out of 368 genes).

**Table 1:** Genes with the highest expression levels in the transcriptomes of the geep and its parents.

Geep vs. *O. aries*	FPKM	Geep vs. *C. hircus*	FPKM	Sheep	FPKM	Goat	FPKM
B2M	24587.1	RPS16	7600.7	RPS11	12435.6	CD74	7886.4
ND4L	13344.8	CRIP1	6533.09	RPS7	12251.6	TMSB10	7085.5
UBB	9910.8	TMSB4X	6246.6	RPS15	10010.5	ACTB	5182.0
ND3	9471.3	RPSA	6005.9	B2M	9144.1	RPS29	4675.6
COX3	8374.3	CD74	5869.0	RPS26	8752.1	RPS8	4496.3
ND4	7581.8	RPLP0	4826.3	RPS8	8487.9	GPX1	4023.1
ATP8	5535.5	RPLP2	4729.0	GNLY	7801.7	RPS7	3782.6
OLA-I	5529.8	RPS7	4671.0	RPS27	7649.8	RPLP0	3749.8
CYTB	5011.0	RPL27	3910.2	UBA52	7042.4	RPS27	3620.3
ATP6	4419.3	RPS15	3200.9	RPLP0	7034.1	TPT1	3538.8

The parental origin of geep transcript was determined before gene expression analysis. Genes of uncertain function were removed.

**Table 2:** By cross-referencing with human data^13^ 645 genes were found to be expressed age-dependent in all three animals.

Pathway	Number of genes	% of all genes	% in humans
Angiogenesis (P00005)	10	1,55	1,21
CCKR signaling map (P06959)	9	1,40	1,26
Apoptosis signaling pathway (P00006)	8	1,24	0,95
Alzheimer disease-presenilin pathway (P00004)	7	1,09	0,63
EGF receptor signaling pathway (P00018)	7	1,09	0,98
FGF signaling pathway (P00021)	7	1,09	0,81
Gonadotropin-releasing hormone receptor pathway (P06664)	7	1,09	1,51
Inflammation mediated by chemokine and cytokine signaling pathway (P00031)	7	1,09	1,56
PDGF signaling pathway (P00047)	7	1,09	1,03
Wnt signaling pathway (P00057)	7	1,09	1,54

Overrepresented pathways in those 645 genes and the corresponding frequencies in humans were calculated.

**Table 3:** Genes expressed in the geep that matched only the paternal or maternal reference genome, cross-referenced with the geneimprint database.

Gene	Origin in geep	Reported expressed allele
AMPD3	paternal	maternal in *Mus musculus*
ATP10A	paternal	maternal in *Homo sapiens*, *Mus musculus* and *Macaca mulatta*
B4GALNT4	maternal	maternal in *Homo sapiens*
CDKN1C	paternal	maternal in *Homo sapiens* and *Mus musculus*
EGFL7	paternal	paternal in *Homo sapiens* (predicted)
GLIS3	maternal	paternal in *Homo sapiens*
GPT	paternal	maternal in *Homo sapiens*
GRB10	maternal	maternal in *Ovis aries*
HSPA6	paternal	maternal in *Homo sapiens* (predicted)
IGF2	paternal	paternal in *Ovis aries*, *Homo sapiens* and *Mus musculus*
KCNQ1	paternal	maternal in *Homo sapiens* and *Mus musculus*
PON1	paternal	maternal in *Homo sapiens*
PRIM2	paternal	Biallelic (conflicting data) in *Homo sapiens*
TH	paternal	maternal in *Mus musculus*

Experimental or predicted results in mammalian species were derived from the geneimprint database (http://www.geneimprint.com, accessed May 2019).

### Variant calling

**Figure 4:**
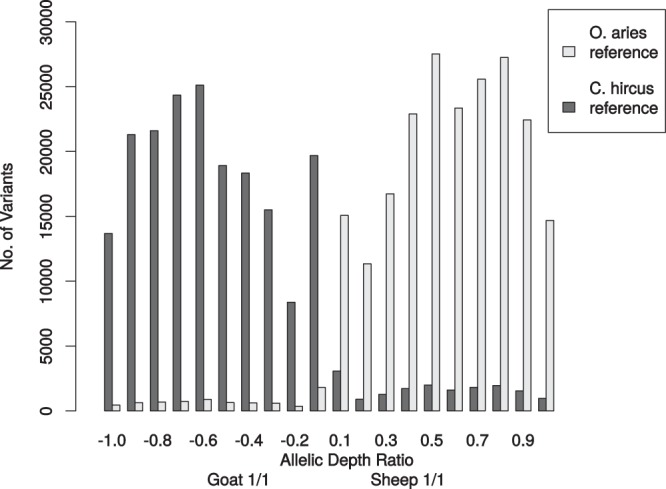
Allelic depth ratio of geep transcript variants where parents show alternative monoallelic expression. Data were sorted into bins of size 0.1, whereas bin 1.0 represents monoallelic expression and bin 0.1 biallelic expression. A positive algebraic sign denotes variants for which the goat expresses the alleles 1/1, bins with a negative algebraic sign contain variants for which the sheep expresses the alleles 1/1.

**Figure 5:**
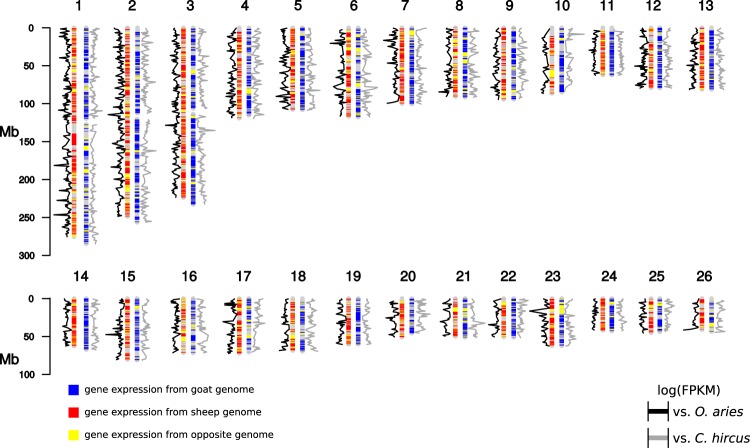
Schematic representation of the geep genome. Origins of transcripts in the geep genome based on variant calling results were plotted on the chromosomes. Red regions denote locations where expression is most likely from the sheep genome, blue indicates expression from the goat genome. Yellow regions are expressed from the genome of the opposite species and do not give information about their actual location on the opposite genome. Gene expression (log of FPKM values) is plotted next to chromosomes. Supplementary Table S10 summarized the contribution of each parent by genomic regions to the geep transcriptome.

**Figure 6:**
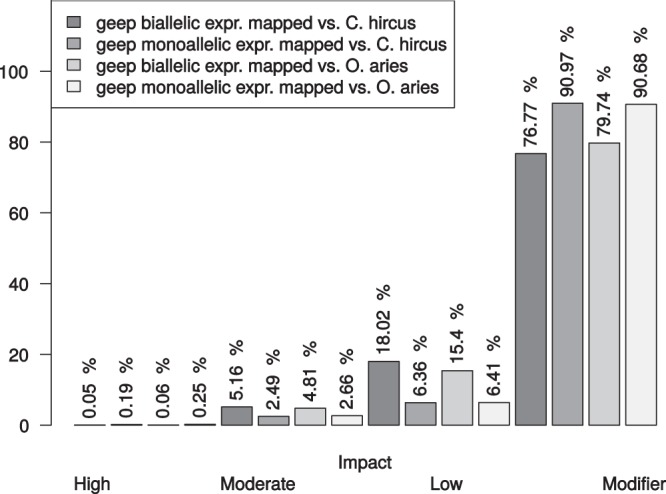
Variant effect prediction of geep variants for which the parents show alternatively monoallelic expression. The percentages of variant impact were quantified for geep transcript variants after partitioning them into mono- and biallelic expression.

The results of the variant calling of all three animals mapped against the two different reference genomes are summarized in Supplementary Table S8. The two variant calling datasets (i) geep, sheep and goat mapped against *O. aries* reference and (ii) geep, sheep and goat mapped against *C. hircus* reference were filtered in order to retain only variants where the two parents had alternative monoallelic expression and for which the geep would consequently have biallelic expression. The transcript-zygosities of the geep for those variants are shown in Supplementary Table S9. The allelic depth ratio for each geep variant was calculated and sorted into bins of size 0.1. An allelic depth ratio of 1 indicates a geep transcript with monoallelic expression (either from sheep or goat) that is supported by all sequencing reads mapped to the respective position whereas an allelic depth of 0 indicates unbiased biallelic expression. Variants where the sheep expressed the alleles 1/1 received a positive algebraic sign and variants where the goat expresses the alleles 1/1 received a negative algebraic sign. The allelic depths of geep variants were plotted against the number of variants in the bins (Fig. 4). In order to elucidate which geep transcripts were dominantly expressed from which parent, variant calls were utilized as described in the material and methods section. For visualization the karyotyping results of our previous study^6^ were used and the genomic positions of dominant transcripts were highlighted on each chromosome (Fig. 5). Variant effect prediction with genes derived from the variant calling revealed that the fraction of moderate and low impact variants is elevated for genes where the parents show alternatively monoallelic variation and the geep shows biallelic expression (Fig. 6). Quantification of geep variants in open reading frames, for which the parents have alternative monoallelic expression, are shown in Supplementary Table S11.

## Discussion

Cases of sheep goat hybrids are very rare with the one presented here being the first reported case of an buck-ewe hybrid^6^. By sequencing RNA from whole-blood samples of the geep and the parental animals, we have the unique opportunity to gather insights into the transcriptomic biology of a hybrid mammal. Since the parent species, *O. aries* and *C. hircus*, are closely related phylogenetically, species discrimination in mapping RNA sequencing reads from the geep was very low with conventional protocols. Hence, four widely used mapping methods were compared: TopHat, Star1pass, Star2pass, and TMAP. All three animals were mapped against the *O. aries* and the *C. hircus* reference genomes to get an estimate of the species discrimination capabilities of the softwares. TMAP was run with the mapall flag, which applies different mapping algorithms in a sequential manner in order to obtain high mapping efficiencies. For a single species approach this is feasible in order to compensate for the comparably high bias of Ion Torrent platforms^14^. In our inter-species approach on the other hand, this mapping strategy led to no species discrimination whatsoever (Fig. 1). TopHat performed best in respect to species discrimination (Fig. 2), but overall mapping efficiencies were comparably low. It is noteworthy that TopHat produced almost no uniquely mapped reads when mapping the goat vs. the *O. aries* genome, which underlines TopHat’s ability to discriminate between species to a certain extent. Star2pass produced the best trade-off between mapping efficiencies and species discrimination (Fig. 2) and was therefore used for all further analyses in the study. Since even TopHat mapped 35–43% of the reads to both reference assemblies we developed a pipeline to acquire uniquely mapped geep sequencing reads and applied it to the Star2pass output. 53.7% of all geep sequencing reads could not be assigned to either genome, which is most likely caused by the small phylogenetic distance between the parents in combination with sequencing errors. Nevertheless, the discovery and quantification of expressed transcripts lead to comparable numbers in all groups (Supplementary Table S1). The parents share 80 transcripts among the 100 highest expressed genes. The slight difference is most likely due to the difference in sex and species and probably age (the exact age of animals at time of sampling is unknown). The fact that the geep only shares 61 genes with the founder animals among the top 100 expressed transcripts can be in part explained by age dependent expression, as discussed below, but might also be an effect of adaptation to the hybrid genome. All genes with a FPKM >1 are summarized in Supplementary Table S2. To clarify to which extent the transcriptomes of the animals differ we used transcripts with a FPKM value >1 and determined the overlap between the four groups (Fig. 3). In total, the geep expressed 219 genes that were not detected in the founders and the founders expressed 1009 genes that were not present in the geep’s transciptome (Supplementary Table S7), which could be age dependent. It was shown in a human twin study that age-related effects on gene expression are highest in blood compared to fat, skin, and lymphoblastoid cell lines^13^. Among those genes is *IGF2*, which we can confirm to stem from the paternal genome as previously described in sheep, humans, and mice^8,10^. *IGF2* expression was solely detected in the geep, which confirms the general conception that expression levels decline with age^15,16^. Due to that finding we cross referenced genes uniquely expressed by the geep or the founder animals with the genes that Viñuela *et al*. found to be expressed in an age dependent manner in human blood^13^. Of the 219 genes uniquely expressed in the geep 88 (40.2%, Supplementary Table S6) are in that dataset. 557 out of 1010 (55.1%, Supplementary Table S6) of the founder genes matched to age-dependently expressed genes in human blood. A pathway analysis of those genes (Table 2) revealed that the same pathways are overrepresented among those genes when compared with age dependently expressed genes in human twin’s blood.

We could also confirm *GRB10*, a gene which is maternally imprinted in sheep, to be expressed from the maternal genome^12^. Cross matching genes from the geep transcriptome, which only mapped to one reference genome with the geneimprint database lead to the discovery of 14 common genes (Table 3). Interestingly, for 9 of those genes the parental origin did not match the database entry. Either this is a property of the taxonomic group (subfamily Caprinae) investigated here, or it is an effect of the hybrid’s unique transcriptome regulation. Experiments in rodent hybrids (crosses of *Peromyscus maniculatis* and *Peromyscus polionotus*) have shown that imprinting patterns in hybrids can drastically deviate from the patterns found in the parental species^17–20^. We propose that the remaining 2,361 genes that the geep shares with only one of each founder are probable candidates for imprinted expression.

Furthermore, 368 genes in the geep transciptome stem from the dam and 870 overlap with the sire transcriptome (Supplementary Table S7). We compared these two groups with a functional annotation clustering analysis (Supplementary Table S5). Gene ontology (GO) terms involving ion binding and regulation of transcription, gene expression and biosynthetic processes are exclusively expressed from the sheep genome. GO terms that are dominantly expressed from the goat are oxidation reduction, enzyme inhibition, inflammatory response and coenzyme binding (Supplementary Table S5). Additional analyses with PANTHER revealed that the gonadotropin-releasing hormone receptor pathway is mainly expressed from the maternal genome, whereas genes involved in inflammation mediated by chemokine and cytokine signaling pathway stem from the paternal genome. Taken together, these findings indicate that the hybrid transcriptome is not a random mixture of the parental transcriptomes, but rather a unique functional entity, which follows imprinting signatures that only partially overlap with sheep or other mammals.

Since a large proportion of the geep’s transcriptome information was lost during the assignment of uniquely mapped reads, we decided to perform variant calling (metrics are summarized in Supplementary Table S8) with the initial sets of reads mapped by Star2pass. A noteworthy difference between the two reference genomes in the variant calling is that the amount of variants in goat vs. *O. aries* is higher compared to geep vs. *O. aries*, whereas sheep vs. *C. hircus* and geep vs. *C. hircus* are in a similar range. A lower number was to be expected in both geep variant call sets. What also caught our attention was the high ratio of heterozygous to homozygous variants in the geep variant call sets compared to the parents. To elucidate to what extent monoallelic variants from the parents are present in the geep transcriptome we used only variants where the founder transcripts are alternatively monoallelic and calculated the allelic depth of those variants in the geep transcriptome (Fig. 4). It became obvious that the expression pattern in the hybrid transcriptome is not fully determined by the alleles expressed in the two parents, i.e. for many transcripts a bias or even monoallelic expression was observed. Although the allelic state of geep transcriptome variants mostly depends on the parental alleles, the allelic depth of geep variants can be considered normally distributed with the exception of bins −0.1 and 0.1, which contain only heterozygous variants. Since the variant calling pipeline considers different factors for the determination of zygosity, like number of reads, mapping quality of reads and base qualities, there is no clear border between hetero- and homozygous calls in Fig. 4. Between 72 and 76% of geep variants, for which the parents express alternatively monoallelic variants, were classified as biallelic by the variant caller (Supplementary Table S9). Since we analyzed transcriptomic, not genomic data, a Mendelian inheritance pattern was not to be expected.

To estimate the genomic regions that contribute dominantly to the geep’s transcriptome, we created an overview of the geep genome, taking the syntenic relationships from the previous study into account^6^, and highlighted stretches that dominantly stem from each of the parents genomes (Fig. 5). In addition, we plotted the gene expression along each chromosome. It became apparent that gene expression and the origin of transcripts based on sequence variants largely correlates, which confirms that both analysis pipelines established in this study produce reliable results. Genomic regions that contain transcripts that were assigned to the goat (blue stretches) sum up to 1,053,846,408 bp and genomic regions that were assigned to the sheep (red stretches) to 913,106,456 bp. Sheep contribution is higher solely on geep chromosomes 6, 17, 20, and 23 (Supplementary Table S10). This confirms our previous finding that a higher number of genes expressed in the geep stem from the *C. hircus* genome (Fig. 3).

Variant effect prediction revealed that variants where the parents are alternatively monoallelic and the geep is monoallelic also have a lower impact. The number of variants belonging to the class “moderate” and “low” are clearly elevated when the geep is biallelic. This indicates that monoallelic expression may be a rescue mechanism to protect from disadvantageous mutations. Another interesting finding is that although the amount of moderate variants is about twice as high for biallelic geep variants the fraction of missense mutations is elevated by about 6% in monoallelic geep variants (Supplementary Table S11). This could indicate that these missense variants might have a rather positive influence on the overall fitness of the hybrid.

With this study, we present the first comprehensive analysis of next generation sequencing data from a mammal hybrid. By developing two pipelines for species discrimination, we were able to draw sensible conclusions about the parental origin of hybrid transcripts and genomic regions. Bioinformatics combined with statistical analyses revealed that this rare buck-ewe hybrid only partially follows imprinting schemes previously described in sheep and other mammals. Furthermore, transcriptome regulation seems to differ from the founder transcriptomes. Taken together these findings lead to the conclusion, that gene and transcriptome regulation in mammal hybrids is distinct from the parental species and is most likely a product of partially incompatible imprinting mechanisms from two closely related species. Together with future studies of this kind, the study presented here could contribute to a deeper understanding of hybridization in evolution. This is especially interesting in respect to human evolution, since Slon *et al*. (2018) demonstrated that hybridization played a role in hominin evolution^21^.

## Material and Methods

### Ethics approval

We used data generated in a previous project. The experimental work has been published by Pauciullo *et al*. (2016) who reported to have conducted the experiments in accordance with German animal welfare legislation and under approval of the institutional committee on the ethics of animal experiments of National Research Council of Italy^6^.

### Animal resources

The female hybrid animal was born under natural conditions in a small flock close to Göttingen (Lower Saxony, Germany). It is the descendant of a male goat (Harzer Ziege) and a female sheep (Leineschaf). A photographic picture of the hybrid is provided as Supplementary Fig. S1.

### Library preparation and sequencing

Blood was isolated and stored with the PAXgene Blood RNA System (BD) and Direct-zol RNA MiniPrep was used for RNA isolation (Zymo Research). RNA quality was determined with the Agilent Bioanalyzer RNA Nano (results summarized in Supplementary Table S12) and library preparation was performed with the Ion Total RNA-Seq kit v2. Quality control of the library was carried out using the Agilent Bioanalyzer DNA 1000 (results summarized in Supplementary Table S13) and qPCR (KAPA Library Quantification Kit Ion Torrent, results summarized in Supplementary Table S14). RNA sequencing was achieved with the template kit Ion PI Hi-Q OT2 200, the sequencing kit Ion PI Hi-Q Sequencing 200 and an Ion PI™ Chip on an Ion Proton platform.

### Alignment

TopHat v. 2.1.0^22^ was run with the following options:–bowtie1–no-novel-juncs–min-isoform-fraction 0.0–min-anchor-length 3 -r 192. Star v. 2.4.2a^23^ was used with default settings. TMAP v. 3.4.0 (https://github.com/iontorrent/TS/tree/master/Analysis/TMAP) was run with the following options: mapall -a 2 -n 8 -v -Y -u -o 1 stage1 map4. RNAseq reads of all three animals were aligned to the sheep (GCF_002742125.1_Oar_rambouillet_v1.0)^24^ and goat (GCF_001704415.1_ARS1)^25^ reference genomes, respectively.

### Discovery of uniquely mapped geep reads

The Star2pass alignment results of the geep reads against both reference genomes were analyzed with cmpBams^26^. Reads that mapped uniquely to a reference genome were extracted. In order to discriminate between reads that mapped to both references the SAM cigar string was used to calculate a score. First the number of matching bases was compared. If that resulted in an equal score the number of insertions and deletions was also considered. If the score was still equal, soft- and hard-clipped bases were included in the scoring. Reads with an equal final score were discarded. The two datasets of unique reads mapped to *C. hircus* or *O. aries* reference genome, respectively were used in the differential expression analysis.

### Transcript quantification and transcriptome comparison

Tanscriptome assembly and determination of transcript expression levels were performed with Cufflinks v. 2.2.1^27^ with default settings apart from–library-type fr-secondstrand. Transcript overlaps between datasets were visualized with the R library VennDiagram. Gene annotation files in general feature (GFF) format were acquired from NCBI. Transcripts with a FPKM value <1 were neglected. Genes with unique official gene symbols (genes of unknown function) were neglected as well since they are not suitable for interspecies comparison.

### Variant calling

For variant calling the Broad Institute workflow #3891 “Calling variants in RNAseq” was followed as closely as possible^28,29^. Known variant datasets used for variant annotation: GCF_000298735.2 (dbSNP build ID 151, source NCBI) and GCA_001704415.1 (dbSNP build ID 143, source Ensemble). Due to the usage of Ion Torrent sequencing data the MarkDuplicates step had to be omitted due to a lack of information provided by the sequencing platform.

### Determination of transcript origin in the geep transcriptome

Variants for the determination of transcript origin were selected as follows: both geep transciptome variant calling datasets (mapped vs. *C. hircus* and mapped vs. *O. aries*) were filtered by variants where the parents are alternatively homozygous and the geep is homozygous. Homozygous reference variants were assigned to be dominantly expressed from the parent that fits the respective reference genome and homozygous alternate variants were classified as dominantly expressed from the opposite founder animal. Sequences of variants along the genome were summarized to blocks for a more intuitive visualization. The resulting data was plotted with the R library chromPlot^30^.

### Variant effect prediction

Variant effects were analyzed using snpEff^31^ with default settings.

### Functional annotation clustering and pathway analyses

Geep transcripts for functional annotation clustering with the Database for Annotation, Visualization and Integrated Discovery (DAVID) v6.7^32^, were taken from the output generated by the R package VennDiagramm. Pathway analyses were performed with the PANTHER (Protein ANalysis THrough Evolutionary Relationships) Classification System^33^.

## Supplementary information

Supplementary Info

Supplementary Dataset

## Data Availability

The raw sequencing data was uploaded to the NCBI Sequence Read Archive (SRA) and is accessible via BioProject ID PRJNA588993.
