# Supplementary material for: Blood transcriptome analysis in a buck-ewe hybrid and its parents
Source: Sci Rep. 2019 Nov 25;9:17492. doi: 10.1038/s41598-019-53901-z (PMC6877586; doi:10.1038/s41598-019-53901-z)
Supplement: Supplementary file 1 — Supplementary Info [file 41598_2019_53901_MOESM1_ESM.pdf]

Supplementary Information for the manuscript

Blood transcriptome analysis in a buck-ewe hybrid and its parents

by

C. Falker-Gieske, C. Knorr, J. Tetens

Supplementary Table S1: Number of discovered transcripts above a FPKM threshold of 1 after mapping of RNA sequencing reads with Star2pass. Geep RNA sequencing reads were mapped against the O. aries and the C. hircus reference genomes and filtered with a species discrimination pipeline. The sheep was mapped against the O. aries reference assembly and the goat was mapped against the C. hircus reference assembly. FPKM values were calculated with Cufflinks.

Supplementary Table S2: Cufflinks results of geep RNAseq reads mapped against O. aries and C. hircus reference genomes with Star2pass and post-mapping species discrimination. Sheep and goat RNAseq reads were mapped against the correspondig genomes.

Supplementary Table S3: Percentage of common genes between animals among the 100 highest expressed genes.

Supplementary Table S4: Intersection of the top 100 expressed genes after removal of genes of uncertain function.

Supplementary Table S5: Functional annotation clustering of geep transcripts, which were uniquely expressed from each founder genome respectively. The gene ontology (GO) terms represent pathways in the geep, which contain only genes from geep/sheep and geep/goat intersections.

Supplementary Table S6: Genes which were only expressed in the geep or it's parents were cross-referenced with genes that have been found to be expressed age-dependent in humans.

Supplementary Table S7: Intersections of expressed genes and genes that were expressed uniquely by the three animals. Transcripts with a FPKM > 1 were analyzed in a Venn diagramm and uniquely expressed genes and relevant intersections were extracted.

Supplementary Table S8: Summary of variant calling metrics. Variant calling on all RNAseq reads mapped with Star2pass were called using the GATK variant calling pipeline from the Broad Institute.

Supplementary Table S9: Quantification of allelic distribution of geep transcript-variants for which the parents showed alternatively monoallelic expression.

Supplementary Table S10: Sums of genomic stretches of parent-contribution to the geep transcriptome in base pairs. Contributions were calculated by using variants for which the parents showed alternative monoallelic expression.

Supplementary Table S11: Variant effects by functional class. Variants in protein coding regions were quantified with respect to their impact. Missense variants lead to an amino acid exchange in the protein, nonsense variants produce nonsense proteins by a frameshift and silent variants have no impact on protein amino acid sequences. Only geep variants for which the parents show alternatively monoallelic expression were used in the analysis and the variants were split by bi- and monoallelic expression.

Supplementary Table 12: Evaluation of RNA quality with an Agilent Bioanalyzer 2100. Electropherogram of each sample shown, respectively.

Supplementary Table 13: Quality control of the sequencing library with an Agilent Bioanalyzer DNA 1000.

Supplementary Table 14: qPCR results obtained with the KAPA Library Quantification Kit Ion Torrent.

Supplementary Figure S1: Photographic picture of the buck-ewe hybrid.

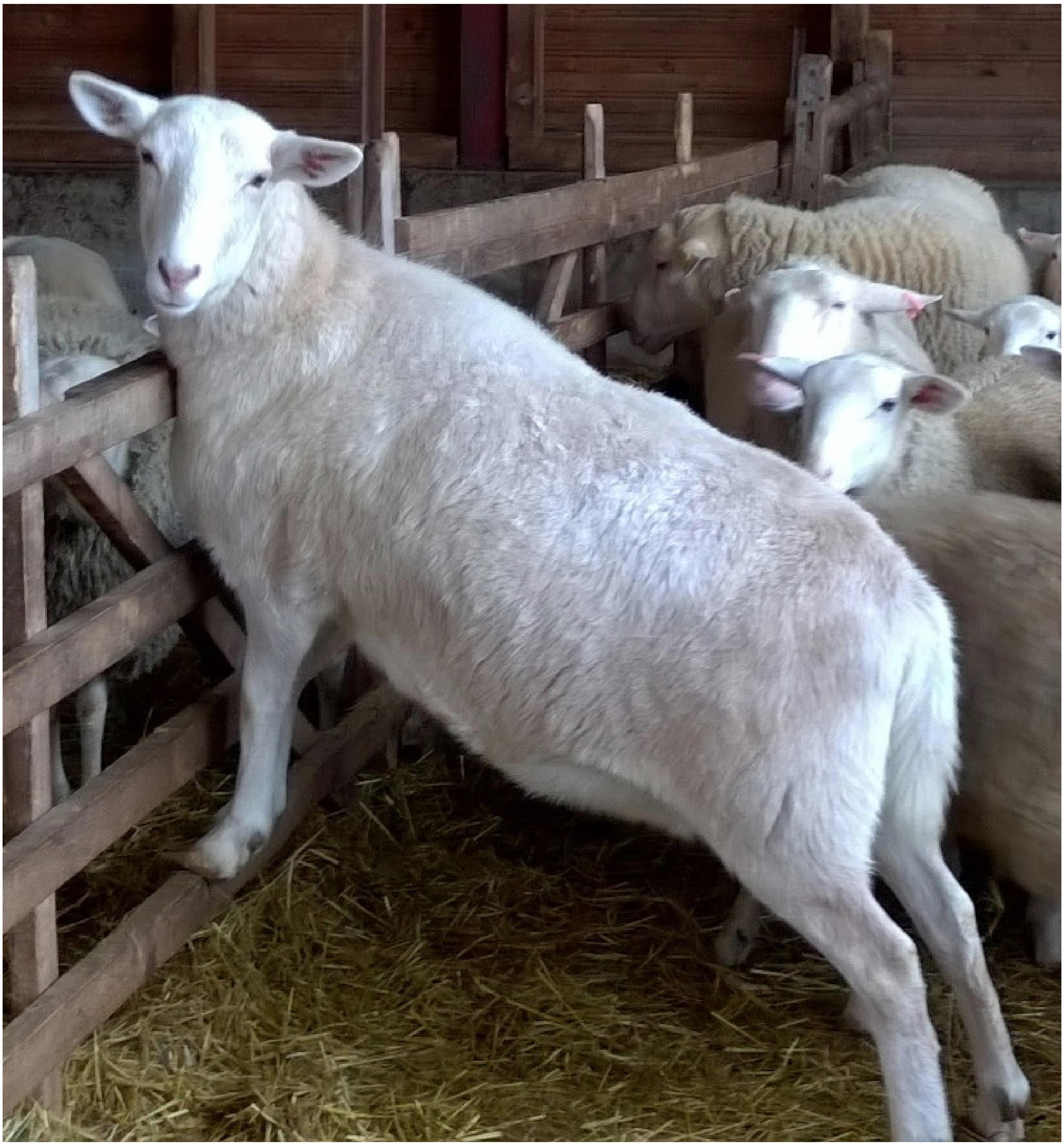

Supplementary Figure S1: Photographic picture of the buck-ewe hybrid.
